# Implications of False Negative and False Positive Diagnosis in Lymph Node Staging of NSCLC by Means of ^18^F-FDG PET/CT

**DOI:** 10.1371/journal.pone.0078552

**Published:** 2013-10-25

**Authors:** Shaolei Li, Qingfeng Zheng, Yuanyuan Ma, Yuzhao Wang, Yuan Feng, Bingtian Zhao, Yue Yang

**Affiliations:** Key Laboratory of Carcinogenesis and Translational Research (Ministry of Education), Thoracic Surgery II, Peking University Cancer Hospital & Institute, Beijing, China; Faculty of Medicine, University of Porto, Portugal

## Abstract

**Background:**

Integrated ^18^F-fluorodeoxyglucose positron emission tomography/computed tomography (^18^F-FDG PET/CT) is widely performed in hilar and mediastinal lymph node (HMLN) staging of non-small cell lung cancer (NSCLC). However, the diagnostic efficiency of PET/CT remains controversial. This retrospective study is to evaluate the accuracy of PET/CT and the characteristics of false negatives and false positives to improve specificity and sensitivity.

**Methods:**

219 NSCLC patients with systematic lymph node dissection or sampling underwent preoperative PET/CT scan. Nodal uptake with a maximum standardized uptake value (SUVmax) >2.5 was interpreted as PET/CT positive. The results of PET/CT were compared with the histopathological findings. The receiver operating characteristic (ROC) curve was generated to determine the diagnostic efficiency of PET/CT. Univariate and multivariate analysis were conducted to detect risk factors of false negatives and false positives.

**Results:**

The sensitivity, specificity, positive predictive value (PPV), negative predictive value (NPV), and accuracy of PET/ CT in detecting HMLN metastases were 74.2% (49/66), 73.2% (112/153), 54.4% (49/90), 86.8% (112/129), and 73.5% (161/219). The ROC curve had an area under curve (AUC) of 0.791 (95% CI 0.723-0.860). The incidence of false negative HMLN metastases was 13.2% (17 of 129 patients). Factors that are significantly associated with false negatives are: concurrent lung disease or diabetes (p<0.001), non-adenocarcinoma (p<0.001), and SUVmax of primary tumor >4.0 (p=0.009). Postoperatively, 45.5% (41/90) patients were confirmed as false positive cases. The univariate analysis indicated age > 65 years old (p=0.009), well differentiation (p=0.002), and SUVmax of primary tumor ≦4.0 (p=0.007) as risk factors for false positive uptake.

**Conclusion:**

The SUVmax of HMLN is a predictor of malignancy. Lymph node staging using PET/CT is far from equal to pathological staging account of some risk factors. This study may provide some aids to pre-therapy evaluation and decision-making.

## Introduction

 Lung cancer is the leading cause of cancer death worldwide and late diagnosis at an advanced stage is a fundamental obstacle to improving lung cancer outcomes. An obvious relationship between TNM stage and survival rate of patients has been shown in some studies [1]. Thus, accurate staging of non-small cell lung cancer (NSCLC) provides important prognostic information and determines the best treatment approach [2]. In particular, metastasis to N2 Lymph nodes is considered to be crucial for operability, while patients without lymph node metastases or only intrapulmonary or hilar lymph node can receive surgery [3]. Neoadjuvant chemotherapy with surgery or concurrent or sequential chemoradiotherapy are legitimate choices for patients with positive N2 lymph nodes [4].

 Even though contrast enhanced CT has been the most common imaging modality for TNM staging, it has limitations in evaluating lymph node status because the prediction of positive lymph nodes on CT is based on size criteria alone [5]. ^18^F-fluorodeoxyglucose positron emission tomography (^18^F-FDG PET) is a functional imaging modality that is based on the increased glucose metabolism of malignant cells [6]. Since the introduction of integrated PET/CT, functional information and morphological information can be combined on TNM staging with ease. Although some previous studies have indicated that the integrated PET/CT are more effective for detecting HMLN metastasis, results regarding the extent of its benefits have been inconsistent [7]. Further, the incidence of occult lymph node metastasis in NSCLC patients showing negative uptake by FDG-PET/CT is 7-16% [8-10], and false positive findings from inflammatory or granulomatous lesions are still problematic on PET/CT.

 The purpose of this study was to assess the diagnostic accuracy of integrated PET/CT in HMLN staging of NSCLC, and to evaluate the characteristics of false negative and false positive lymph nodes to improve specificity and sensitivity.

## Materials and Methods

### Patient Selection and Staging

 A review was undertaken for NSCLC patients who underwent surgery from January 2010 to January 2013 at the department of Thoracic Surgery Ⅱ, Peking University Cancer Hospital. All patients who received neoadjuvant chemotherapy and patients with bulky mediastinal node metastases before thoracotomy were excluded. The remaining 219 consecutive patients with histologically proven NSCLC underwent staging with integrated PET/CT prior to lung resection. Over 90% of 219 patients underwent systematic lymph node dissection and the other less 10% patients underwent systematic lymph node sampling. 129 patients who were staged HMLN negative and 90 patients HMLN positive by PET/CT underwent lung resection with systematic lymph node dissection or sampling. The participants signed informed consent before PET/CT scan which included a statement to approve their disease information could be used in one study. This study has been approved with the Ethics Committee of Beijing Cancer Hospital. Disease stage was evaluated, according to the TNM Classification of Malignant Tumors, 7th Edition.

### Integrated PET/CT Scan

 PET/CT was performed using a Gemini TF PET/CT system (Philips). All patients fasted for at least 6h before the PET/CT scan and only glucose-free water was allowed. An intravenous injection of 3.7 MBq of ^18^FDG/kg of body weight was administered and patients rested for 60min before scanning. PET/CT data was obtained from patients in the supine position. Emission imagines were acquired after CT scanning, and an emission scan was performed in 8-10 bed positions with 1 min per step. The FDG uptake of tumor was visually compared with that of the surrounding tissue in areas devoid of prominent artifacts and overlapping increased FDG uptake organs. A team of experienced radiologists reviewed the integrated PET/CT images independently. Nodal uptake with a SUVmax >2.5 was interpreted as positive. All integrated PET/CT imaging was performed within 4 weeks of surgery.

### Surgical Resection and Pathological Examination

 All of the surgical resections and nodal dissections were conducted by thoracic surgeons at the department of Thoracic Surgery Ⅱ of Peking University Cancer Hospital. Systematic lymph node dissection or sampling was carried out in stations 2R, 4R, 3A, 3P, 7-12 in right-sided tumors, and in 4L, 5, 6, 7-12 for left-sided tumors. The remaining of N1 nodes (13,14) were removed as part of the resection specimen. All resected tumor specimens were examined by experienced pulmonary pathologists. Histological classification of NSCLC was based on the WHO classification. The dissected lymph nodes were examined histologically following hematoxylin and eosin staining.

### Statistical Analysis

 The results of PET/CT were compared with the histopathological findings. Subsequently, sensitivity, specificity, PPV, NPV, and accuracy were determined by diagnostic test fourfold table. Additionally, a ROC curve was generated by SPSS Statistics 17.0 software package (SPSS Inc.) to determine the cut-off SUVmax for detecting HMLN metastases. Univariate data analysis was conducted using Pearson’s chi-square test. Multivariate analysis was conducted using the logistic regression (backwards stepwise) method. P-values were considered statistically significant at p<0.05.

## Results

### Evaluation of HMLN status by pathological examination and PET/CT

 The characteristics of the enrolled patients are shown in Table 1. Of these 219 patients, 175 (79.9%) had adenocarcinoma, 32 (14.6%) had squamous cell carcinoma, and 12 had other types including 5 adenosquamous carcinomas, 2 large cell carcinomas, 2 carcinoids, and 2 mucoepidermoid carcinomas, 1 hemangioendothelioma. Positive HMLN was found in 30.1% (66/219) of those patients. A total of 1173 hilar and mediastinal lymph stations (2959 lymph nodes) were dissected and 140 (11.9%) stations showed positive. 

**Table 1:** Characteristics of patients and tumors (n=219).

Characteristics	Distribution(n)
Sex	
Male/Female	120/99
Age	
Mean±SD/Range	60.7±11.1/26-86
Smoking status	
Smoker/Never Smoker	106/113
History of lung disease or diabetes	
Yes/Never	30/189
Location of the tumor	
Central/Non-central	48/171
Lobar distribution of the tumor	
RUL/RML/RLL/LUL/LLL*	82/15/37/52/33
Histopathological type	
Adenocarcinoma/Squamous cell carcinoma/Other types	175/32/12
Pleural invasion	
Yes/No	104/115
Pathological T stage	
T1a/T1b/T2a/T2b/T3/T4	43/40/113/5/11/7
Lymph node metastasis	
pN0/pN1/pN2	145/25/49
Pathological stage after surgery	
Ⅰa/Ⅰb/Ⅱa/Ⅱb/Ⅲa/Ⅲb/Ⅳ	67/62/26/7/42/3/12
Tumor size (cm)	
Median/Mean±SD/Range	2.6/2.8±1.4/0.5-11.0
SUVmax of primary tumor	
Median/Mean±SD/Range	5.4/6.2±4.1/0-24.2

* RUL: right upper lobar; RML: right middle lobar; RLL: right lower lobar; LUL: left upper lobar; LLL: left lower lobar

**Figure 1:**
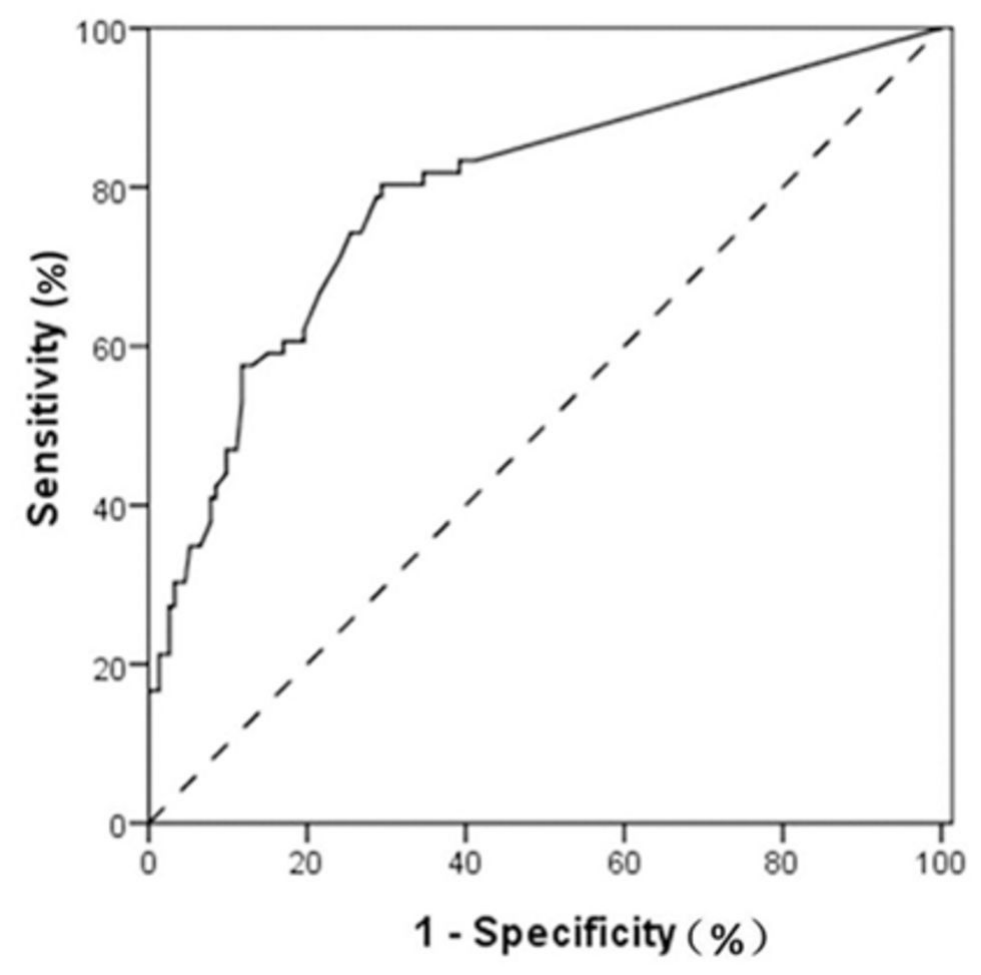
ROC curve for SUVmax of HLMN. The curve had an AUC of 0.791 (95% CI 0.723-0.860) with a cut-off SUVmax of 2.34.

 All evaluating indicators of PET/CT examination were analyzed in Table 2. The sensitivity, specificity, PPV, NPV, and accuracy of PET/ CT in detecting HMLN metastases were 74.2% (49/66), 73.2% (112/153), 54.4% (49/90), 86.8% (112/129), and 73.5% (161/219). The ROC curve based on the SUVmax of lymph nodes is shown in Figure 1. The SUVmax of nodes had an AUC of 0.791 (95% CI 0.723-0.860) with a cut-off SUVmax of 2.34. If nodal uptake with SUVmax >2.34 were interpreted as positive, the sensitivity, specificity, PPV, NPV, and accuracy of PET/ CT were 80.3% (53/66), 70.6% (108/153), 54.4% (53/98), 86.8% (108/121), and 73.5% (161/219).

**Table 2:** Histopathological findings and PET/CT results of HMLN.

	SUVmax ≦ 2.5	SUVmax >2.5
Pathological negative	112 (TN)	41 (FP)
Pathological positive	17 (FN)	49 (TP)

TN, true negatives; FP, false positives; FN, false negatives; TP, true positives.

### The patterns and risk factors of HMLN metastasis of SUVmax ≦ 2.5

 The incidence of occult HMLN metastasis in this study was 13.2% (17 of 129 patients). Of 17 patients with lymph node metastasis, multistation metastasis was found in 9 patients (52.9%), while the other 8 patients showed on a single station (47.1%). The table 3 details the preoperative PET/CT scanning and the patterns of HMLN involvement. 13 of 17 patients had metastasis in hilar lymph node. 5 patients had concurrent diabetes although they had normal levels of fasting blood glucose before the PET/CT scan. Three patients had concurrent pneumonia or tuberculosis. 11 patients had not even a little bit FDG uptake of lymph node, also the nodal uptakes of other 6 patients with SUVmax ≦ 2.5 were interpreted as negative.

**Table 3:** Pattern of false negative HMLN involvement (17 of 129 patients).

Case	Lobar distribution	Concurrent disease	Histopathological type	Tumor size(cm)	SUVmax of tomor	SUVmax of node	Pathological N(+) station
1	RUL	None	Squamous cell carcinoma	2.8	7.3	2.4	10
2	RUL	None	Adenosqumaous carcinoma	2.4	12.8	0	2R,4R
3	RUL	None	Adenocarcinoma	1.4	7.0	0	10
4	RUL	None	Adenocarcinoma	2.3	6.0	2.4	10
5	RUL	Tuberculosis	Adenocarcinoma	1.6	3.0	0	10
6	RML	None	Squamous cell carcinoma	3.5	8.8	0	10
7	RLL	None	Adenocarcinoma	4.0	12.0	2.5	2R,4R,7,10
8	RLL	Pneumonia	Adenocarcinoma	2.1	3.3	0	10
9	LUL	diabetes	Adenosqumaous carcinoma	3.0	5.4	0	4L,6,10
10	LUL	diabetes	Adenocarcinoma	3.6	4.4	1.7	6,10
11	LUL	None	Adenocarcinoma	2.8	10.2	2.5	4L,5,6,7,10
12	LUL	diabetes	Adenosqumaous carcinoma	2.5	5.4	0	4L,6,10
13	LLL	diabetes	Adenocarcinoma	3.9	4.7	0	7
14	LLL	None	Adenocarcinoma	3.1	4.5	0	7
15	LLL	None	Large cell carcinoma	3.4	5.3	0	4L,6,7,10
16	LLL	diabetes	Squamous cell carcinoma	4.4	7.6	2.2	4L,6,7,9
17	LLL	Tuberculosis	Hemangioendothelioma	1.5	2.4	0	5,7,10

Table 4 summarized the results of univariate analysis for factors associated with HMLN metastasis in 129 PET/CT negative patients. Factors that were significantly associated with HMLN metastasis were: concurrent lung disease or diabetes (p<0.001), non-adenocarcinoma (p<0.001), and SUVmax of primary tumor > 4.0 (p=0.009). The multivariate risk-factor analysis (Table 5) identified concurrent lung disease or diabetes, non-adenocarcinoma, pleural effusion, and SUVmax of primary tumor > 4.0 as risk factors for false negative HMLN metastasis.

**Table 4:** Univariate analysis for factors associated with HMLN metastasis in 129 PET/CT negative patients.

Variable	Pathologically positive	Pathologically negative	P-value
Sex			
Male	9	59	
Female	8	54	0.929
Age			
≦65	14	75	
>65	3	37	0.201
Smoking status			
Never smoker	11	62	
Smoker	6	50	0.469
Concurrent lung disease or diabetes			
Yes	8	9	
Never	9	103	0.000
Location of the tumor			
Central	5	16	
Non-central	12	96	0.115
Lobar distribution			
Upper or middle lobe	10	79	
Lower lobe	7	33	0.331
Right or left lung distribution			
Right lung	8	78	
Left lung	9	34	0.066
Histopathological type			
Adenocarcinoma	9	98	
Non-adenocarcinoma	8	14	0.000
Differentiated degree			
High	1	25	
Medium	10	63	
Lower	6	24	0.200
Pleural invasion			
Yes	11	46	
No	6	66	0.067
Tumor size			
≦3cm	10	81	
>3cm	7	31	0.255
SUVmax of primary tumor			
≦4.0	3	58	
>4.0	14	54	0.009

**Table 5:** Multivariate analysis for factors associated with HMLN metastasis in 129 PET/CT negative patients.

Variable	Odds ratio	Confidence interval	P-value
Concurrent lung disease or diabetes	10.34	2.43-38.76	0.001
Non-adenocarcinoma	3.76	0.98-15.31	0.052
Pleural invasion	3.55	0.95-13.02	0.060
SUVmax of primary tumor >4.0	3.25	0.89-17.06	0.072

### The analysis of risk factors associated with false positive detection of HMLN metastasis of SUVmax > 2.5

90 patients who had the SUVmax of MHLN >2.5 were diagnosed as positive cases by PET/CT. Postoperatively, 41 patients were confirmed as false positive cases. The univariate analysis indicated age >65 years old (p=0.009), well differentiation (p=0.002), and SUVmax of primary tumor ≦4.0 (p=0.007) as risk factors for false positive uptake. Risk factors that were significantly associated with false positive uptake in multivariate analysis were well differentiation (p=0.069), non-adenocarcinoma (p=0.007), and age >65 years old (p=0.001). We displayed some details of the results in Table 6 and Table 7.

**Table 6:** Univariate analysis for factors associated with false positives in 90 PET/CT positive patients.

Variable	Pathologically positive	Pathologically negative	P-value
Sex			
Male	30	23	
Female	19	18	0.623
Age			
≦65	36	19	
>65	13	22	0.009
Smoking status			
Never smoker	23	17	
Smoker	26	24	0.603
Concurrent lung disease or diabetes			
Yes	7	6	
Never	42	35	0.963
Location of the tumor			
Central	16	11	
Non-central	33	30	0.548
Lobar distribution of the tumor			
Upper or middle lobe	33	27	
Lower lobe	16	14	0.881
Right or left lung distribution of the tumor			
Right lung	26	22	
Left lung	23	19	0.955
Histopathological type			
Adenocarcinoma	40	28	
Non-adenocarcinoma	9	13	0.142
Differentiated degree			
High	0	6	
Medium	17	21	
Lower	32	14	0.002
Pleural invasion			
Yes	28	19	
No	21	22	0.307
Tumor size			
≦3cm	27	26	
>3cm	22	15	0.425
SUVmax of primary tumor			
≦4.0	7	16	
>4.0	42	25	0.007

**Table 7:** Multivariate analysis for factors associated with false positives in 90 PET/CT positive patients.

Variable	Odds ratio	Confidence interval	P-value
medium differentiation(vs. low)	2.51	0.93-6.80	0.069
Non-adenocarcinoma	5.28	1.58-17.65	0.007
Age > 65	6.18	2.05-18.63	0.001

## Discussion

 The determination of HMLN metastasis is an important part of staging in patients with NSCLC. Stereotactic body radiation therapy (SBRT) is not recommended for small peripheral lung cancer patients with hilar lymph node involved (N1) [11]. When contralateral mediastinal lymph nodes (N3) or more than one ipsilateral mediastinal lymph nodes are involved, primary surgery is not recommended and such patients are commonly treated with chemotherapy and radiotherapy. In recent years, PET/CT is widely used for the staging of NSCLC [12], which is expected to be a very promising optimization tool for lymph nodal staging. In a meta-analysis [7], the pooled sensitivity and specificity on a per-patient analysis were 71.9% and 89.8%, respectively. It suggested PET/CT had more specificity but less sensitivity for lymph nodal staging. So we undertook the present study to improve the sensitivity and specificity of integrate PET/CT.

The results of our study can be summarized as follows: The SUVmax of HMLN is a predictor of malignancy. The ROC curve identified optimal cut-off SUVmax of 2.34 for HMLN metastasis in our cohort. The false negatives and false positives accounted for 13.2% (17/129) patients and 45.6% (41/90), respectively.

 In previous study [13], as in this study, a traditional value for the SUVmax of 2.5 was chosen. HMLN with a value of 2.5 or greater was considered positive. Integrated PET/CT provides somewhat unsatisfied sensitivity (74.2%), specificity (73.2%), and reasonably low accuracy (73.5%) for nodal staging of NSCLC (on a per-patient basis). In order to maximize the accuracy of integrated PET/CT we generate ROC curve. The curve identifies the optimal cut-off SUVmax value that maximizes the sensitivity and specificity and consequently the accuracy. The value found was 2.34. When a SUVmax of 2.34 or greater is used to label any lymph node as positive on PET/CT and less than 2.34 as negative, the sensitivity of all patients is 80.3% at the expense of specificity. The accuracy in SUVmax of 2.34 is remarkably similar to the accuracy reported in SUVmax of 2.5. In ROC curve analysis, it is obvious that with increasing of cut-off value of SUVmax, the specificity become higher and higher at the expense of sensitivity. When SUVmax reach at value of 3.85 or greater, the specificity is at least 90.0%, while the sensitivity is only 47.0%. 

Gerfolio [10,14] has shown the clinical importance and relevance of the SUVmax of a primary non-small cell lung tumor. It provides information on a tumor’s biologic aggressiveness, key pathologic features, and its potential to spread. This study illustrates the importance of the SUVmax of lymph nodes. The SUVmax of the primary tumor along with each lymph node that is hypermetabolic should be provided by nuclear radiologist in the text of every PET/CT report.

 Seventeen of one hundred twenty nine patients had falsely negative nodes from integrated PET/CT, who had microscopic positive disease. 11 of 17 patients had N2 disease, and 8 of 11 patients had more than one N2 lymph node station involved. This finding was concordant with comparable previous studies. Al-Sarraf [8] reported the incidence of occult N2 disease in similar patients as 16%, while Cerfolio [15] reported a 14% incidence. When they limited the analysis to clinical stage i patients, the incidence decreased to 7% [9].

Risk factors for occult N2 disease reported in those previous studies were as follows: adenocarcinoma, tumors located in right upper lobe, larger tumor size, a high SUVmax of the primary tumor, centrally located tumors, positive N1 nodes on PET and poorly differentiated histology. A limitation of these studies was the lack of histological examination of involved lymph nodes. It is similar to previous studies that a high SUVmax (>4.0) of the primary tumor was also identified as risk factor for false negatives in the present study.

In our study, visceral pleural invasion (VPI) was identified as a risk factor for occult lymph node metastasis in both univariate and multivariate analysis. One possible reason [16] is that the visceral pleura is very rich in lymphatic vessels, with an intercommunicating “network” arranged over the lung surface and penetrating into the lung parenchyma to join the bronchial lymph vessels with drainage to various hilar lymph nodes. The larger lymphatic vessels in the visceral pleura have one-way valves which also direct flow toward the hilum of the lung. VPI was significantly associated with more extensive HMLN involvement. The present findings support the suggestion by Shimizu et al. that there is a possible VPI tumor cell pathway through the subpleural lymphatics, hilar lymph nodes, and into the mediastinal lymph nodes [17]. 

Interestingly, we found concurrent lung disease or diabetics and non-adenocarcinoma were also risk factors for occult nodal metastasis, although Kubota [18] insisted on that the accuracy of PET/CT were not affected as long as the blood glucose level in diabetic patients was controlled at less than 140mg/dL and Kanzaki [19] thought occult lymph node metastasis more likely to occur in patients of lung adenocarcinoma. Obviously, it showed a distinct trend in this study that there are five diabetic patients of 17 false negative cases. By the way, it is reported that lymph node metastases from adenocarcinoma were of normal size (the short-axis diameter≤ 1 cm) more frequently than those from squamous cell carcinoma [5]. Therefore, we speculate that patients with lymph node metastasis from squamous cell carcinoma tended to be excluded in Kazaki’s series and included in our series of patients.

PET/CT imaging is the most accurate imaging modality for lung cancer staging but faces the most serious challenge due to increased glycolytic activity of benign tumors and inflammatory tissue, in addition to that of malignant tumors. As expected, up to 45.5% patients (41/90) were confirmed as false positives with upstaging from N0 to N1 or N2 by PET/CT scan. These false-positive results may have been caused by reactive hyperplasia or active inflammation due to granulomatous diseases such as tuberculosis [20]. Risk factors for false positives in the present study were old age, well differentiation, low SUVmax of primary tumor and non-adenocarcinoma. Therefore, we still recommend biopsies of all suspicious lymph nodes with a high SUVmax which should not be equated with malignancy until tissue confirmation is obtained. 

Our study has several limitations. Firstly, it suffers from selection bias, because we included patients that received surgical nodal dissection. Therefore, many more cases of early stage of NSCLCs were included, which may have contributed to observed sensitivity reduction. Secondly, it was inevitable that false negative findings are calculated on relatively small numbers. In addition, the analysis was based on a per-patient basis rather than on a per-node station basis.

In conclusion, the SUVmax of HMLN is a predictor of malignancy. The PET/CT had the best diagnostic efficiency at the cut-off SUVmax of 2.34 in our cohort. False negatives and false positives were inevitable but predictable according to some risk factors. Lymph node staging of PET/CT is far from equal to pathological staging.
